# Comparison of glycopyrronium versus tiotropium on the time to clinically important deteriorations in patients with COPD: a post-hoc analysis of randomized trials

**DOI:** 10.1038/s41533-018-0084-8

**Published:** 2018-05-24

**Authors:** Anthony D’Urzo, Giovanni Bader, Steven Shen, Pankaj Goyal, Pablo Altman

**Affiliations:** 1 Department of Family and Community Medicine, Faculty of Medicine, University of Toronto, Toronto, Canada; 2 Novartis Pharma AG, Fabrikstrasse 2, Basel, Switzerland; 3 Novartis Pharmaceuticals Corporation, East Hanover, NJ USA

## Abstract

Glycopyrronium is a once-daily, inhaled long-acting muscarinic antagonist (LAMA) demonstrating similar efficacy to inhaled tiotropium in patients with moderate-to-severe COPD; however, the benefit of LAMAs on COPD symptoms has been variable. COPD is a progressive disease in which many patients develop an acute or sustained deterioration. Data on the prevention of clinically important deteriorations (CID) using LAMAs are limited. A pooled analysis was performed on four Phase III trials (*n* = 2936) that compared the efficacy of glycopyrronium (*n* = 1859) with tiotropium (*n* = 1077). The primary endpoint was significant delay and/or reduction in the occurrence of CID. CID was defined as any of the following: ≥100 mL decrease from baseline in pre-dose forced expiratory volume in 1 second (FEV_1_), ≥4 point increase in St George’s Respiratory Questionnaire score or a moderate-to-severe COPD exacerbation occurring after the first dose of study medication. A sustained CID was a CID occurring on ≥2 consecutive visits 4 weeks apart or for ≥50% of all available subsequent visits. Baseline characteristics for the overall population were similar. Patients had moderate (62%) or severe (38%) COPD. Mean post-bronchodilator FEV_1_ was approximately 55% predicted, and mean FEV_1_ reversibility was 16.7 and 18.6% in the glycopyrronium and tiotropium groups, respectively. Both glycopyrronium and tiotropium significantly reduced time to CID and sustained CID versus placebo (*p* < 0.001). No statistically significant differences were found between the glycopyrronium and tiotropium treatment groups in time to CID or sustained CID. Glycopyrronium is effective in delaying time to clinically important deteriorations, with similar efficacy to tiotropium.

## Chronic lung disease: Potential alternative treatment option

A novel inhaled drug shows promise in treating moderate to severe chronic lung disease. Guidelines recommend long-term treatment of chronic obstructive pulmonary disease (COPD) with long-acting muscarinic antagonist (LAMA) inhalers. Tiotropium is a common COPD LAMA. Now, Anthony D’Urzo at the University of Toronto, Canada, together with an international research team, have carried out a pooled analysis of results from four clinical trials to compare the efficacy of a novel LAMA, glycopyrronium, with tiotropium. In these trials, 2936 patients were treated with either glycopyrronium or tiotropium, and the health status of patients using glycopyrronium was also compared with those given a placebo. Glycopyrronium was as effective as tiotropium in delaying or preventing clinically important deteriorations in patient health. The risk of deteriorations was significantly lower in the glycopyrronium group than the placebo group.

## Introduction

Chronic obstructive pulmonary disease (COPD) is a heterogeneous condition comprising multiple pulmonary and extra-pulmonary manifestations.^1,2^ The disease has a variable natural history and significant heterogeneity exists with respect to clinical presentation, response to therapy, and survival.^1–3^ As a result, there is consensus that spirometry alone does not adequately reflect the complexity of the disease and is an incomplete marker of the severity of symptoms, exercise limitation and health status.^3,4^

In recognition of the complexities of COPD, a large number of guideline-based therapies are available that aim to improve symptoms, reduce the frequency and severity of exacerbations, and improve health status and exercise tolerance.^5^ The Global Initiative for Chronic Obstructive Lung Disease (GOLD) 2017 strategy now recommends that COPD management should consider both disease impact (i.e., assessment of symptoms) measured by the COPD Assessment Test (CAT) or modified Medical Research Council (mMRC) dyspnea scale, and exacerbation history.^5^ Guidelines recommend long-acting bronchodilator therapy, including long-acting muscarinic antagonists (LAMAs), as a first choice therapy in all GOLD patient groups.^5^

Glycopyrronium is a once-daily LAMA indicated for the maintenance treatment of patients with COPD.^6^ Phase III trials have shown that glycopyrronium produces rapid and sustained improvements in lung function, symptoms, health status, exercise endurance and exacerbation risk in patients with COPD.^7–11^ Although glycopyrronium has an efficacy and safety profile similar to the widely prescribed LAMA tiotropium bromide,^7,9,11,12^ studies have indicated a faster onset of action and greater bronchodilation with glycopyrronium within the first 4 h after the first dose on Day 1 of treatment.^9^

COPD clinical trials primarily focus on bronchodilator response using minimal clinically important difference (MCID) to determine improvements in lung function and patient-reported outcomes.^13–15^ Clinically important deterioration (CID) assesses individual deteriorations in lung function, health status and moderate-to-severe exacerbations.^15^

The efficacy and safety of glycopyrronium has been evaluated in a number of randomized controlled trials, primarily in patients with moderate-to-severe COPD. We conducted a post-hoc analysis of four Phase III trials, namely the glycopyrronium bromide in COPD airways (GLOW1, GLOW2, GLOW5)^9–11^ and SHINE studies,^7^ to compare the efficacy of glycopyrronium and tiotropium in patients with COPD in terms of CID delay and/or reduction.

## Results

### Study populations and baseline characteristics

A total of 2936 patients were included in this analysis (glycopyrronium *n* = 1859; tiotropium *n* = 1077). Baseline characteristics were similar for the two treatment groups (Table 1). For the overall population, the mean age was 64 years, the majority of patients were male (74% and 72% in the glycopyrronium and tiotropium groups, respectively), and the mean duration of COPD was 6.5 years. Most patients in both treatment groups had either moderate (62%) or severe (38%) COPD. Although the incidence of cardiac comorbidities was low in the overall population, approximately 25% of patients had documented hypertension and approximately 6% of patients had type 2 diabetes mellitus. Overall, there were no meaningful differences between treatment groups for spirometry measurements at baseline (Table 1). Mean post-bronchodilator forced expiratory volume in 1 second (FEV_1_) was approximately 55% of predicted and mean FEV_1_ reversibility was 16.7% and 18.6% in the glycopyrronium and tiotropium groups, respectively. Few patients had a history of an exacerbation (25% of patients reported a COPD exacerbation within the previous 12 months; Table 1). A risk calculation based on the GOLD strategy was therefore not performed on the overall population as it was considered that the calculation would not be representative of patients with different exacerbation phenotypes.

**Table 1:** Baseline characteristics in the overall population

Characteristic	Glycopyrronium (*N* = 1859)	Tiotropium (*N* = 1077)
Male (*n*; %)	1379 (74.18)	775 (71.96)
Age (years; SD)	63.9 (8.92)	63.7 (8.40)
BMI (kg/m^2^; SD)	26.5 (5.96)	26.6 (5.76)
Smoking status		
Ex-smoker (*n*; %)	1105 (59.44)	621 (57.66)
Current smoker (*n*; %)	754 (40.56)	455 (42.29)
Duration of COPD, years (SD)	6.5 (5.93)	6.5 (5.73)
Exacerbation in previous 12 months (*n*; %)	449 (24.15)	272 (25.26)
Concomitant COPD medication (*n*; %)		
Short-acting anticholinergic	576 (30.98)	357 (33.15)
Long-acting anticholinergic	180 (9.68)	97 (9.01)
Short-acting beta-agonist	268 (14.42)	186 (17.27)
Long-acting beta-agonist	760 (40.88)	483 (44.85)
Inhaled corticosteroids	1015 (54.60)	594 (55.15)
Combination LABA/ICS	647 (34.80)	422 (39.18)
Combination LABA/LAMA	180 (9.68)	109 (10.12)
Xanthine	167 (8.98)	91 (8.45)
SGRQ total score (SD)	48.0 (17.66)	48.2 (17.49)
BDI total score (SD)	6.2 (2.17)	6.3 (2.06)
Severity of airflow limitation (*n*; %)		
GOLD 1 (mild)	2 (0.11)	1 (0.09)
GOLD 2 (moderate)	1148 (61.75)	661 (61.37)
GOLD 3 (severe)	701 (37.71)	414 (38.44)
GOLD 4 (very severe)	8 (0.43)	0 (0.00)
Comorbidities (*n*; %)		
Coronary artery bypass graft	10 (0.54)	5 (0.46)
Myocardial infarction	29 (1.56)	32 (2.97)
Stroke	21 (1.13)	14 (1.30)
History of hypertension	352 (18.93)	366 (33.98)
Type 2 Diabetes mellitus	93 (5.00)	86 (7.99)
FEV_1_, L (SD)		
Pre-bronchodilator	1.3 (0.47)	1.3 (0.48)
Post-bronchodilator	1.5 (0.49)	1.5 (0.49)
FEV_1_, % Predicted (SD)		
Pre-bronchodilator	48.0 (13.44)	47.3 (13.45)
Post-bronchodilator	54.9 (13.14)	55.0 (13.14)
FVC, L (SD)		
Pre-bronchodilator	2.8 (0.83)	2.8 (0.82)
Post-bronchodilator	3.1 (0.89)	3.1 (0.85)
FEV_1_/FVC, % (SD)		
Pre-bronchodilator	48.1 (10.89)	47.4 (10.89)
Post-bronchodilator	49.2 (10.63)	48.9 (10.69)
FEV_1_, Reversibility (%; SD)	16.7 (15.46)	18.6 (15.80)
FVC, Reversibility (%; SD)	13.6 (14.45)	14.8 (15.56)
Blood eosinophil count (cells/µL) (SD)	2.7 (1.84)	2.7 (1.76)

Data are mean (SD) or n (%) unless otherwise stated

*BDI* baseline dyspnea index, *BMI* body mass index, *COPD* chronic obstructive pulmonary disease, *FEV*_*1*_ forced expiratory volume in 1 second, *FVC* forced vital capacity, *GOLD* Global Initiative for Chronic Obstructive Lung Disease, *ICS* inhaled corticosteroid, *LABA* long-acting beta-agonist, *LAMA* long-acting muscarinic agonist, *o.d.* once daily, *SD* standard deviation, *SGRQ* St George’s Respiratory Questionnaire

### Efficacy

#### First and sustained CID

**Fig. 1:**
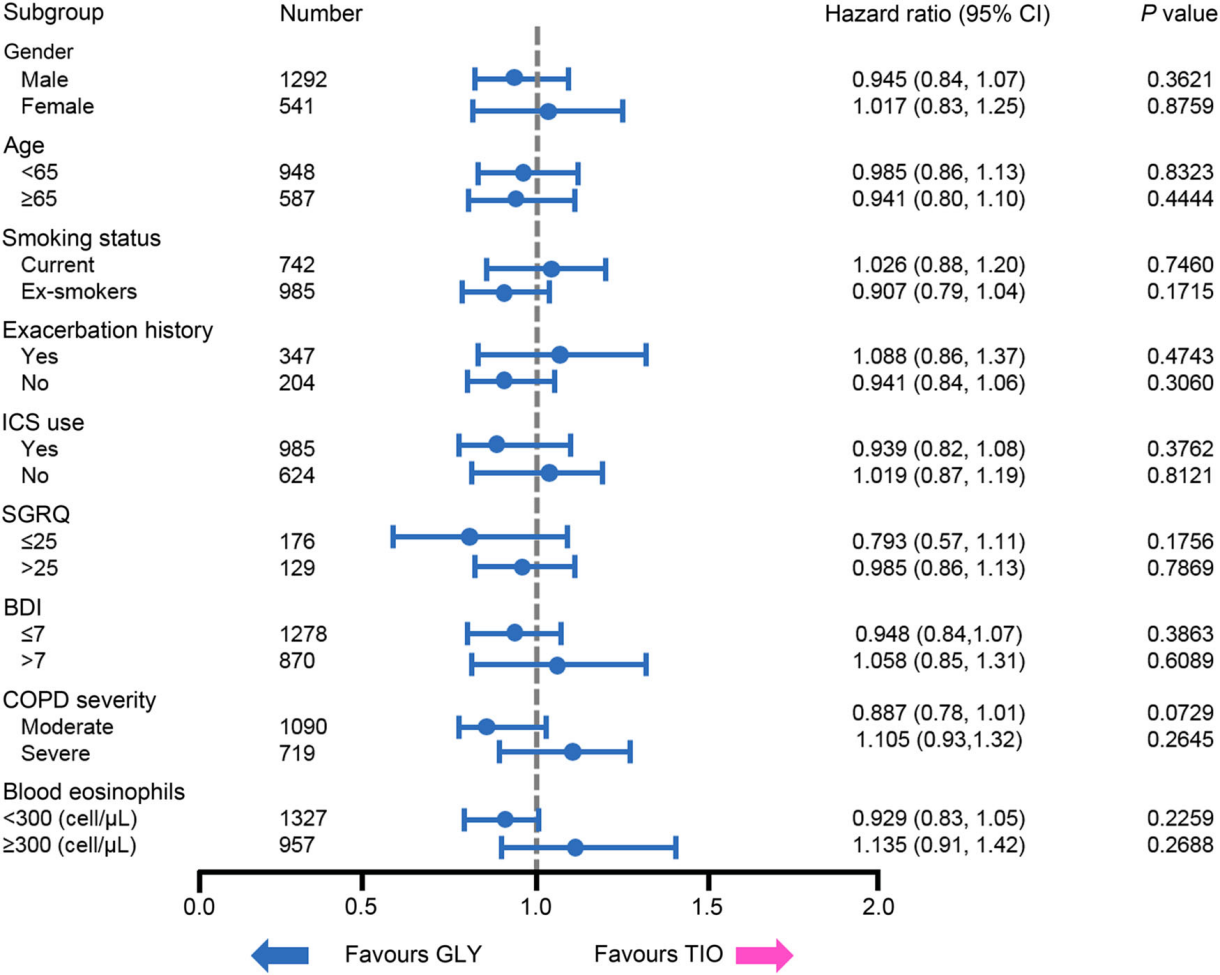
Clinically important deteriorations (CID): glycopyrronium versus tiotropium subgroup analysis. Forest plot depicting the results of a subgroup analysis which assessed the risk of experiencing a clinically important deterioration with glycopyrronium (GLY; *n* = 1859) treatment compared with tiotropium (TIO; *n* = 1077), based on gender, age, smoking status, exacerbation history, inhaled corticosteroid (ICS) use, baseline St. George’s Respiratory Questionnaire (SGRQ), Baseline Dyspnea Index (BDI) score, COPD severity or blood eosinophil levels. Hazard ratios ±95% confidence intervals (CI) are shown

**Fig. 2:**
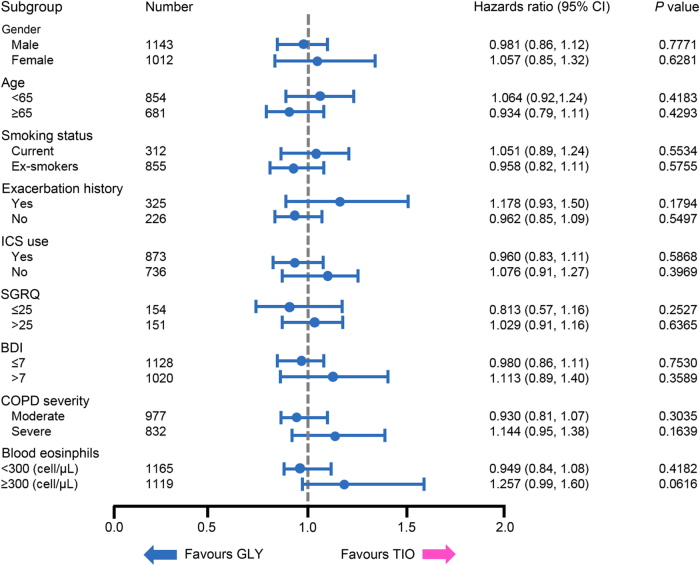
Sustained clinically important deteriorations: glycopyrronium versus tiotropium subgroup analysis. Forest plot depicting the results of a subgroup analysis which assessed the risk of experiencing a sustained clinically important deterioration with glycopyrronium (GLY; *n* = 1859) treatment compared with tiotropium (TIO; *n* = 1077), based on gender, age, smoking status, exacerbation history, inhaled corticosteroid (ICS) use, baseline St. George’s Respiratory Questionnaire (SGRQ), Baseline Dyspnea Index (BDI) score, COPD severity or blood eosinophil levels. Hazard ratios ±95% confidence intervals (CI) are shown

The proportion of patients who experienced a CID ranged non-significantly from 57.8% in the tiotropium group to 60.1% in the glycopyrronium group (*p* = 0.4658; Table 2). This study showed that there was no significant difference in the number of glycopyrronium patients who experienced a sustained CID compared with those receiving tiotropium (53.6% versus 50.2%, *p* = 0.9765; Table 2). The most frequently experienced individual component of the CID definition was a ≥100 mL decline from baseline in FEV_1_ across the glycopyrronium (38.5%) and tiotropium (37.5%) treatment arms (*p* = 0.4850). This was also true for sustained CIDs, where 28.6% of glycopyrronium and 27.5% of tiotropium patients experienced a lung-function-related sustained CID (*p* = 0.5764). There were also no significant differences between the proportion of patients in either treatment group who experienced a St George’s Respiratory Questionnaire (SGRQ)-related CID or sustained CID (*p* = 0.1658 and *p* = 0.4702, respectively), or an exacerbation-related CID or sustained CID (*p* = 0.1735 and *p* = 0.1735, respectively) (Table 2). Subgroup analysis demonstrated that the risk of experiencing a CID or sustained CID was not significantly different between the glycopyrronium and tiotropium groups, regardless of gender (male or female), age (<65 or ≥65 years), smoking status (current or ex-smoker), exacerbation history, inhaled corticosteroid (ICS) use (yes or no), baseline SGRQ (≤25 or >25) or Baseline Dyspnea Index (BDI; ≤7 or >7) score, COPD severity (moderate or severe) or blood eosinophil level (<300 or ≥300 cells/μL) (*p* > 0.0729; Figs. 1 and 2). Of note, glycopyrronium significantly reduced the risk of both first and sustained CID compared with placebo across all subgroups (*p* < 0.001) (Supplementary Figs. 1 and 2).

**Table 2:** Number of patients with first and sustained clinically important deteriorations in the glycopyrronium and tiotropium treatment groups

Deterioration	Glycopyrronium (*N* = 1859)	Tiotropium (*N* = 1077)	*P* value
**First CID**	1117 (60.1)	622 (57.8)	0.4658
FEV_1_	715 (38.5)	404 (37.5)	0.4850
SGRQ	432 (23.2)	249 (23.1)	0.1658
Exacerbation	390 (21.0)	194 (18.0)	0.1735
**Sustained CID**	996 (53.6)	541 (50.2)	0.9765
FEV_1_	531 (28.6)	296 (27.5)	0.5764
SGRQ	104 (5.6)	64 (5.9)	0.4702
Exacerbation	390 (21.0)	194 (18.0)	0.1735

Values are expressed as *n* (%)

*CID* clinically important deteriorations, *FEV*_*1*_ forced expiratory volume in 1 second, *SGRQ* St. George’s Respiratory Questionnaire

#### Time to first and sustained CID

**Fig. 3:**
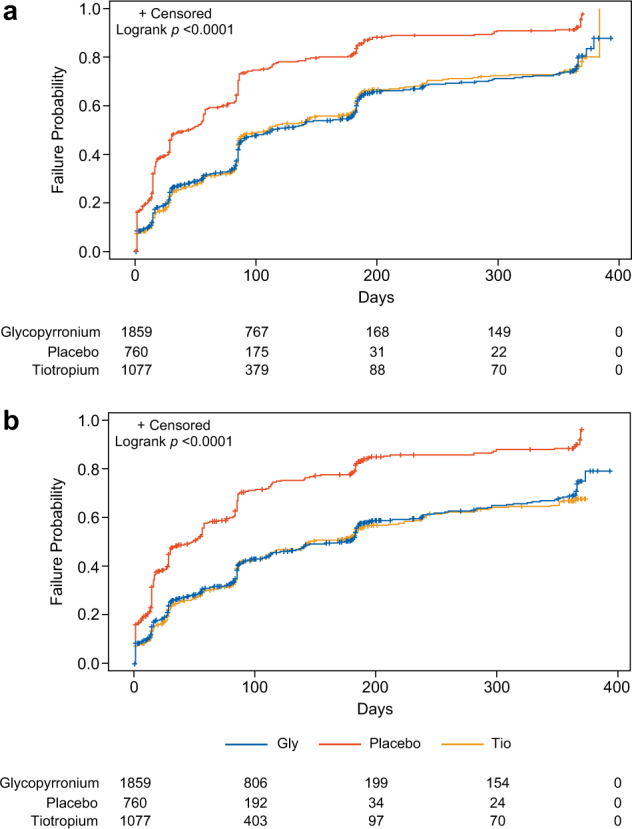
Kaplan–Meier time to clinically important deteriorations (CID): glycopyrronium versus tiotropium and placebo. Kaplan–Meier graph depicting the time to **a** first or **b** sustained CID in patients receiving glycopyrronium (blue line), tiotropium (yellow line), and placebo (orange line)

Neither the time to first CID nor sustained CID in patients receiving glycopyrronium was significantly different compared with tiotropium (HR for CID: 0.96 [95% CI: 0.87, 1.07], *p* = 0.4658 and HR for sustained CID: 1.00 [95% CI: 0.90, 1.12], *p* = 0.9765). Glycopyrronium (*n* = 1859) significantly reduced time to first CID and sustained CID compared with placebo (*n* = 760) (HR: 0.46 [95% CI: 0.41, 0.51] and HR: 0.44 [95% CI: 0.40, 0.49], both *p* < 0.0001) (Fig. 3a, b).

#### FEV_1_ and SGRQ at first and sustained CID

At the time of first CID, there was no difference in mean FEV_1_ ± SD (1.29 ± 0.466 L versus 1.32 ± 0.501 L, *p* = 0.3452) or mean SGRQ ± SD score (53.92 ± 16.87 units versus 57.74 ± 17.47 units, *p* = 0.3903) in patients who had been receiving glycopyrronium compared with tiotropium respectively. Similar non-significance was also observed for these parameters at the time of sustained CID (Table 3).

**Table 3:** Comparison of FEV_1_ and SGRQ at baseline and at first and sustained clinically important deterioration between the glycopyrronium and tiotropium treatment groups

Deterioration	Glycopyrronium	Tiotropium	*P* value
**First CID**			
FEV_1_ (L)	*N* = 715	*N* = 404	
At baseline	1.47 ± 0.481	1.50 ± 0.527	0.3045
At time of first CID	1.29 ± 0.466	1.32 ± 0.501	0.3452
SGRQ (Units)	*N* = 432	*N* = 249	
At baseline	43.42 ± 16.791	43.33 ± 16.928	0.9497
At time of first CID	53.92 ± 16.87	57.74 ± 17.47	0.3903
**Sustained CID**			
FEV_1_ (L)	*N* = 531	*N* = 296	
At baseline	1.50 ± 0.482	1.51 ± 0.534	0.7356
At time of sustained CID	1.33 ± 0.480	1.33 ± 0.499	0.9184
SGRQ (Units)	*N* = 104	*N* = 64	
At baseline	43.09 ± 16.914	43.11 ± 18.328	0.9947
At time of sustained CID	54.02 ± 17.5	52.36 ± 18.18	0.5617

Data are presented as mean ± standard deviation

*FEV*_*1*_ forced expiratory volume in 1 second, *SGRQ* St George’s Respiratory Questionnaire, *CID* clinically important deterioration, *N* number of patients

## Discussion

This post-hoc analysis compared the effect of once-daily glycopyrronium with tiotropium on the delay or reduction in the time to first CID and sustained CID in patients with COPD. The results of this analysis demonstrated that the proportions of patients who experienced either a CID or sustained CID were comparable between patients treated with glycopyrronium and tiotropium, and that the risk of experiencing a CID or sustained CID was significantly reduced with glycopyrronium therapy compared with placebo. Subgroup analysis further supported these results.

In this study, a CID was defined as one or more of the following over the 12-week study period: a ≥100 mL decrease from baseline in pre-dose FEV_1_, a ≥4 point increase in SGRQ total score from baseline and/or a moderate-to-severe COPD exacerbation occurring after the first dose of the study medication. A sustained CID was defined as the occurrence of a CID on ≥2 consecutive study visits 4 weeks apart, or for ≥50% of all subsequent study visits. Of note, the majority of CIDs reported in this study relate to a decrease in lung function. The clinical relevance of a CID or, perhaps more importantly a sustained CID, on the progression or burden of COPD remains to be elucidated, but it is likely that the long-term consequences of these events are under-appreciated at present.^15^ Although supporting data is currently lacking, it is possible that the use of a composite index such as CID or sustained CID may be more sensitive for detection of response to treatment, compared with a single measured outcome such as FEV_1_.^16^

Glycopyrronium is a LAMA that has been shown to provide rapid and sustained improvements in lung function, symptoms and health status in patients with moderate-to-severe COPD.^7–11^ Previous studies evaluating the efficacy of glycopyrronium versus tiotropium have demonstrated comparable efficacy with respect to improvements in spirometric endpoints (with the exception of faster onset of action in favor of glycopyrronium), TDI focal score and SGRQ total score.^7,9,11^ The duration of action of once-daily glycopyrronium (50 μg) and tiotropium (18 μg) has been shown to be comparable. In the GLOW5 study, once-daily glycopyrronium was shown to be equally as effective as tiotropium at maintaining FEV_1_ 24 h post-dose.^9^ Furthermore, data from the GLOW2 study demonstrated similar comparability between glycopyrronium and tiotropium for post-dose FEV_1_ AUC_0–24h_ at Week 12 of the study.^11^

The CAT was not available in the analyzed studies, and instead the SGRQ was used to provide the health status/health-related quality-of-life measurements. SGRQ scores have been shown to be reproducible and sensitive to treatment effects over extended time periods,^4,17^ and correlate well with CAT scores (*r* = 0.84).^18^ BDI and TDI were initially developed to help to address the limitations associated with mMRC. These indices provide a multidimensional and comprehensive assessment of breathlessness and are helpful in the evaluation of impact of therapies.^19,20^ Of note, glycopyrronium and tiotropium were comparable in terms of their impact on health status, and their impact on CID or sustained CID was unaffected by BDI (≤7 or >7).

Some limitations of this current analysis should be considered when interpreting the results. Although the studies included in this pooled analysis included similar populations and were of similar design, the study durations varied between 12 and 52 weeks. Data from the GLOW5 study were captured at Week 12, which may not have been sufficiently adequate to observe notable changes in lung function or symptom-related outcomes. In addition, exacerbation burden was shown to be low in the overall population, which precluded our ability to conduct a meaningful risk calculation. Although similar definitions of a CID as described herein have been employed previously,^15,16,21^ the fact that any strong correlation between a CID and other well-established markers of disease progression (e.g., FEV_1_ decline) is yet to be demonstrated, is a potential limitation in this study. Furthermore, the data presented in this post-hoc analysis were collected from four individual clinical trials which were not originally designed to assess CID.

Taken together, the results of this post-hoc analysis demonstrate that glycopyrronium is effective in delaying/reducing time to CIDs, with similar efficacy to tiotropium. This is an important consideration as over half of the patients studied in this analysis experienced at least one CID. Current COPD guidelines recommend that health status and exacerbation history should be considered in disease assessment.^5^ CIDs encompassing these parameters can provide new insight into the management of COPD, which might help reduce disease progression and individualize patient care particularly with regards to lung function, health status and exacerbations. Further studies, with a larger population size and longer follow-up, will be required to confirm these findings. The long-term impact of CID on COPD disease progression also remains to be studied. Finally, the results presented here suggest that glycopyrronium may be an alternative LAMA option for patients with COPD.

## Methods

This post-hoc analysis was carried out using lung function, health status, and exacerbation data from 2936 patients in four large, multicenter, randomized clinical trials conducted in patients with moderate-to-severe airflow obstruction, namely the GLOW1, GLOW2, GLOW5^9–11^ and SHINE studies,^7^ which had similar study populations (Supplementary Table 1). These studies were registered with clinical trials.gov as NCT01005901, NCT00929110, NCT01613326 and NCT01202188 respectively. A fifth study, the SPARK study, also compared glycopyrronium with tiotropium for lung function, health status and exacerbation outcomes.^22^ However, as SPARK was conducted in a different COPD patient population (patients with severe to very severe airflow obstruction), it was decided not to include data from SPARK in this analysis. All available data (captured at the end of each respective study) were included. The exacerbation data used within this post-hoc analysis were captured at the time at which the exacerbation occurred during the entire duration of the studies. GLOW5 was a 12-week study; the FEV_1_, and SGRQ data were captured from this study every 4 weeks up to Week 12. GLOW1 and SHINE were both 26-week studies, and the data from these studies were collected every 4 weeks up to week 26. Lastly, the GLOW2 study was conducted over a 52-week period, and the FEV_1_, and SGRQ data were collected from this study every 4 weeks up to Week 52.

### Patients

Inclusion criteria were age ≥40 years with a smoking history of ≥10 pack-years and moderate-to-severe stable COPD [defined as post-bronchodilator FEV_1_ ≥30–<80% predicted, and post-bronchodilator FEV_1_/forced vital capacity (FVC) ratio <0.70].

### Clinically important deterioration

The primary endpoint of this study was the delay and/or reduction in occurrence of CIDs with once-daily glycopyrronium 50 µg (delivered via the Breezhaler^®^ device; *N* = 1859) compared with once-daily tiotropium 18 µg (delivered via the HandiHaler^®^ device; *N* = 1077) in the GLOW1, GLOW2, GLOW5 and SHINE studies. Tiotropium was prescribed as open-label in GLOW2 and SHINE. Secondary endpoints included evaluation of breathlessness measured using the TDI and health status according to the SGRQ. The duration of the four individual studies included in this analysis ranged from 12 to 52 weeks, and all available data were included in the analyses.

CID was defined as ≥1 of the following components at the post-baseline visit:

- a 100 mL decrease from baseline in pre-dose FEV_1_^13,23^
- ≥4 point increase in SGRQ total score from baseline^14^
- a moderate-to-severe COPD exacerbation occurring after the first dose of the study medication^15^

Time to CID was the time to the first event of any of the above three components. A sustained CID for this analysis was defined as a CID occurring on two or more consecutive visits 4 weeks apart or for ≥50% of all available subsequent visits.^24^ COPD exacerbations were defined as worsening of two or more major symptoms (dyspnea, sputum volume or sputum purulence) for at least two consecutive days or worsening of any one major symptom together with any minor symptom (colds, fever without other cause, increased cough, increased wheeze or sore throat) for at least two consecutive days.^9^

The GOLD strategy recommends the use of the CAT or mMRC dyspnea scale for classifying patients into the “low-symptom” or “high-symptom” groups.^5^ As these measures were not employed in the GLOW or SHINE studies, SGRQ total score and BDI were used as surrogate measures for health status and breathlessness respectively, in addition to baseline lung function (FEV_1_ <50% and ≥50%).

The SGRQ is designed to measure impact on overall health, daily life, and perceived well-being in patients with obstructive airways disease and has become the most widely documented measure of health status in COPD trials.^5,25^ Scores range from 0 to 100, with higher scores indicating more limitations.^25^ Earlier studies have demonstrated that SGRQ and CAT scores are closely correlated,^26–28^ with a CAT score of 10 being comparable to a SGRQ total score of ≥25.^5,26^ As CAT scores were not reported in the GLOW or SHINE glycopyrronium studies, it was not included in the current analysis. The BDI has previously been shown to correlate with mMRC grading for dyspnea assessment.^20,29–32^ BDI cut-off scores of ≥7 and <7 were used in the subgroup analyses of this study as a surrogate for mMRC grades 0–1 and ≥2, respectively. The cut-off value of 7 was chosen since this is the median of the 13-point BDI scale and similar cut-off values were used in another post-hoc analysis of pooled data from trials where BDI was used as a surrogate for mMRC.^33^

### Statistical analyses

All available patients in the full analysis set (FAS) were included in this pooled analysis, and their data were captured at the end of their respective studies (12 to 52 weeks) for use in this analysis. As this was a post-hoc analysis, no power or sample size justification was considered.

These analyses assessed the effect of glycopyrronium versus tiotropium, and glycopyrronium versus placebo on significant delay and/or reduction in the occurrence of CID and sustained CID. The significance level of two-sided 0.05 was used to evaluate the statistical significance of treatment comparisons, and the significant impact of the covariates on the time to CID and sustained CID. Data analysis was performed using SAS software Version 9.13. Due to the large sample size (2936 patients), the normality assumption of the data was deemed sufficient to be analyzed by ANCOVA/mixed effect model.

The summary statistics (*N* number and percentage) of CIDs are included, and the Kaplan–Meier Curves with log rank tests are presented for pooled glycopyrronium versus tiotropium, and versus placebo, results. The median times with 95% confidence intervals, (25% and 75% quartiles) of the time to event are presented. The Cox proportional hazard model was used, with treatment group, gender, age group, baseline COPD severity, smoking status (Yes/No), and high (≥300) or low (<300) baseline eosinophil status, as treatment comparisons.

If a patient did not meet the criteria for a deterioration during the study, the patient was censored at the end of the study for the time to event analysis. Patients who had no event or discontinued early from the study, were censored from the analysis at the last contact date of the study.

The subgroups defined below were used in the subgroup analyses of the time to CID or sustained CID:

- Male versus female
- (GOLD A and B) and (GOLD C and D)^34^ at baseline
- Low and high BDI: ≤7 and >7
- Low and high SGRQ: ≤25 and >25
- Age <65 and ≥65 years at baseline
- Ex- versus current smokers at baseline
- Subjects classified as high (≥300) or low (<300) eosinophils at baseline
- ICS use at baseline (Yes/No)
- History of any exacerbation in previous year before randomization (Yes/No)

### Code availability

Any codes used in the analyses of these data are available from the authors.

### Data availability

The current post-hoc analyses were performed based on primary data from four studies. These primary data have been published previously,^7,9–11^ and result summaries have been posted on the Novartis clinical trial database (https://www.novartisclinicaltrials.com/TrialConnectWeb/home.nov) and other online public databases. Given the post-hoc nature of the data discussed in the present manuscript, these have not been made available on any publicly-accessible database. More information on Novartis’ position on access to clinical trial results and patient-level data is available here: https://www.novartis.com/our-science/clinical-trials/clinical-trial-information-disclosure.

### Ethical approval

These studies were individually approved by the relevant ethics committee and institutional review board at the participating centers at the time of their undertaking. The current post-hoc analysis used anonymized data from these studies, and hence ethics committee or institutional review board approvals were not necessary.

### Informed consent

All participants provided written informed consent to take part in the GLOW1, GLOW2, GLOW5 and SHINE studies.

## Electronic supplementary material

Supplementary Appendix
