# Supplementary material for: Comparison of glycopyrronium versus tiotropium on the time to clinically important deteriorations in patients with COPD: a post-hoc analysis of randomized trials
Source: NPJ Prim Care Respir Med. 2018 May 24;28:18. doi: 10.1038/s41533-018-0084-8 (PMC5967309; doi:10.1038/s41533-018-0084-8)
Supplement: Supplementary file 1 — Supplementary Appendix [file 41533_2018_84_MOESM1_ESM.pdf]

Supplementary Information

Comparison of glycopyrronium versus tiotropium on the time to clinically important deteriorations in patients with COPD: a post-hoc analysis of randomized trials

Anthony D'Urzo, Giovanni Bader, Steven Shen, Pankaj Goyal, Pablo Altman

SUPPLEMENTARY FIGURES

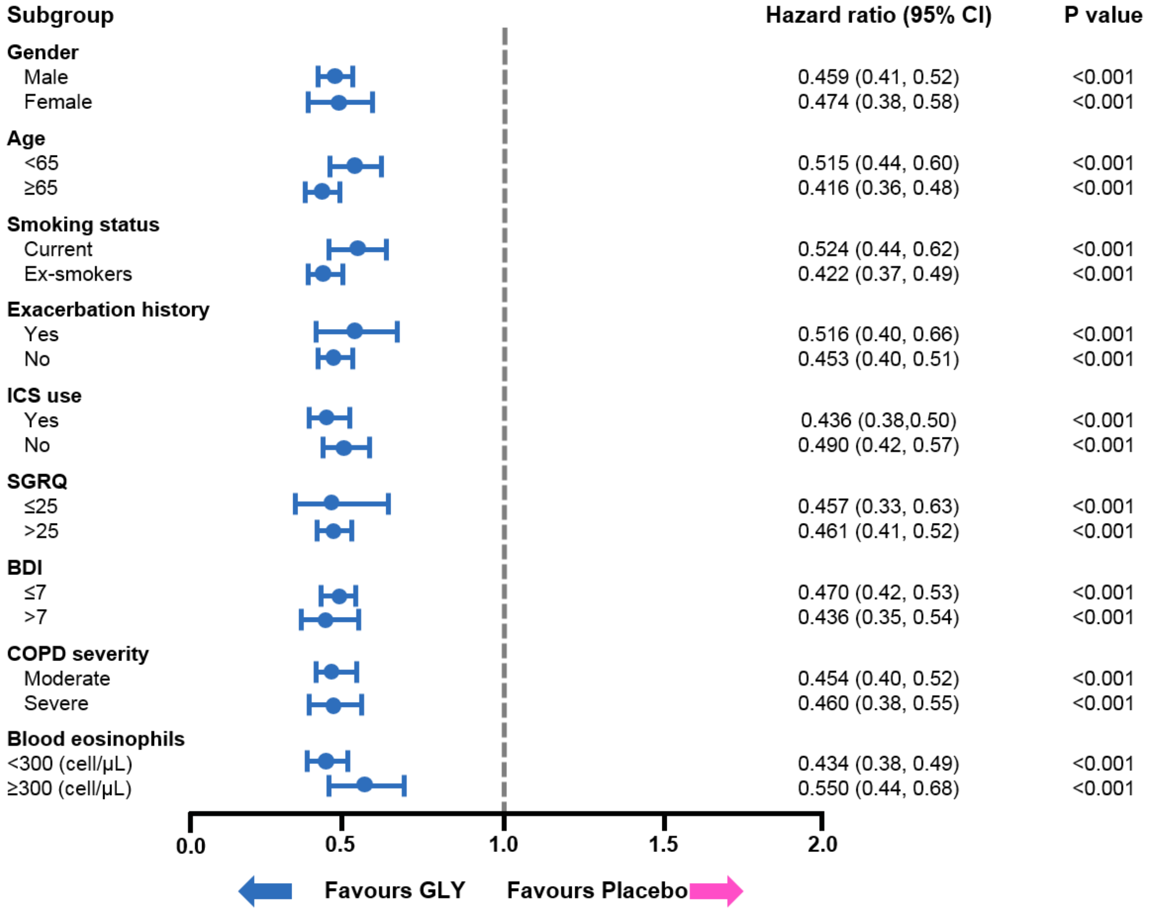

Supplementary Figure 1: Clinically important deteriorations (CID): glycopyrronium versus placebo subgroup analysis

Forest plot depicting the results of a subgroup analysis which assessed the risk of experiencing a clinically important deterioration with glycopyrronium (GLY) treatment (n=1859) compared with placebo (n=760), based on gender, age, smoking status, exacerbation history, inhaled corticosteroid (ICS) use, baseline St. George's Respiratory Questionnaire (SGRQ), Baseline Dyspnea Index (BDI) score, COPD severity or blood eosinophil levels. Hazard ratios ± 95% confidence intervals (CI) are shown.

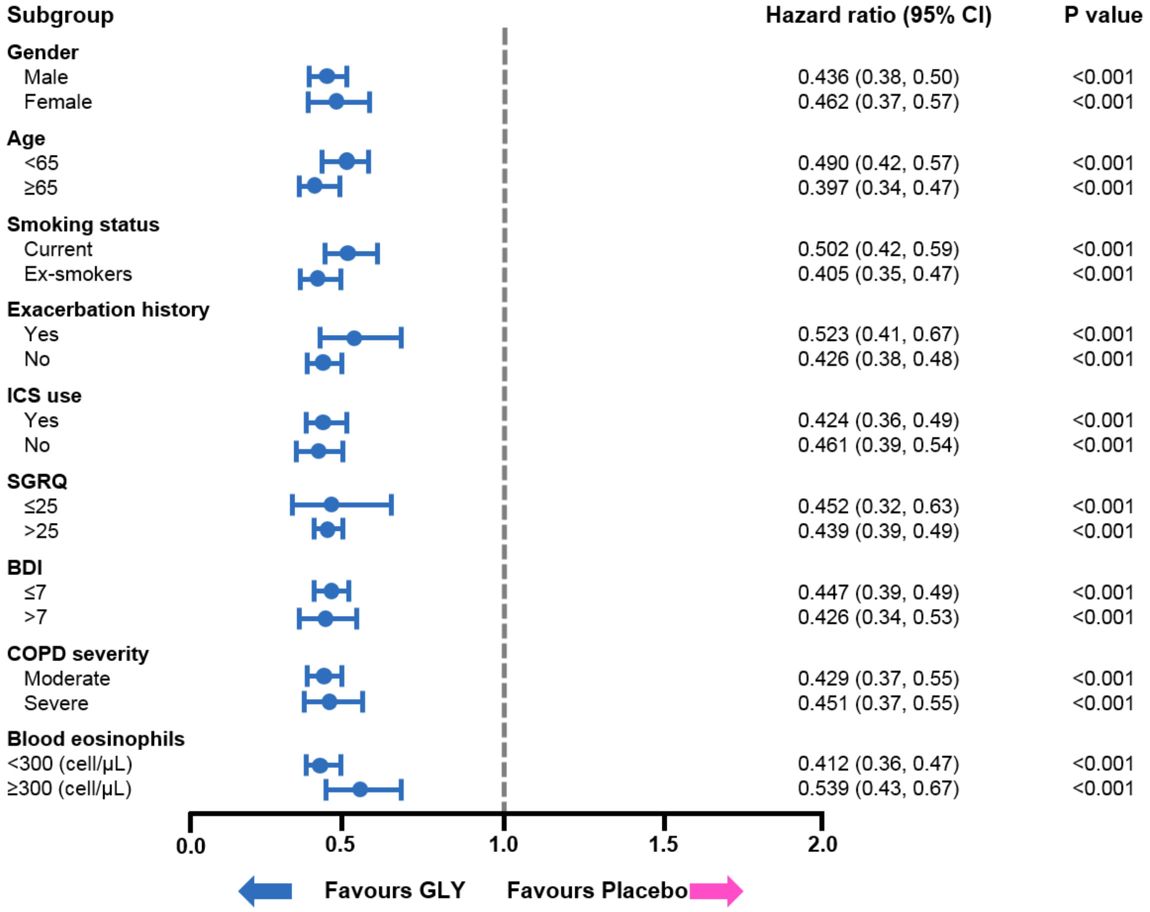

Supplementary Figure 2: Sustained clinically important deteriorations (CID): glycopyrronium versus placebo subgroup analysis

Forest plot depicting the results of a subgroup analysis which assessed the risk of experiencing a sustained clinically important deterioration with glycopyrronium (GLY) treatment (n=1859) compared with placebo (n=760), based on gender, age, smoking status, exacerbation history, inhaled corticosteroid (ICS) use, baseline St. George's Respiratory Questionnaire (SGRQ), Baseline Dyspnea Index (BDI) score, COPD severity or blood eosinophil levels. Hazard ratios ± 95% confidence intervals (CI) are shown.

SUPPLEMENTARY TABLES

Supplementary Table 1: Study design of trials included in the post-hoc analysis

Study	Duration and population size	Treatments	Key inclusion criteria	Endpoints
GLOW1 (D'Urzo et al. 2011: Study number NCT01005901)	26 weeks<br>N=822	Glycopyrronium 50 µg od<br>Placebo	• Aged ≥40 years<br>• Smoking history ≥10 pack-years<br>• Moderate-to-severe COPD (post-bronchodilator FEV_1 ≥30% and <80% predicted; post-bronchodilator FEV_1/FVC ratio <0.70)	Primary endpoint:<br>• Trough FEV_1 at Week 12<br>Key secondary:<br>• Change in TDI focal score and SGRQ total score at Week 26<br>• Time to first moderate or severe COPD exacerbation<br>• Daily rescue medication use over 26 weeks
GLOW2 (Kerwin et al. 2012: Study number NCT00929110)	52 weeks<br>N=1066	Glycopyrronium 50 µg od<br>Placebo<br>Tiotropium 18 µg od (open-label)	• Aged ≥40 years<br>• Smoking history ≥10 pack-years<br>• Moderate-to-severe stable COPD (post-bronchodilator FEV_1 ≥30% and <80% predicted; post-bronchodilator FEV_1/FVC ratio <0.70)	Primary endpoint:<br>• Trough FEV_1 at Week 12<br>Key secondary:<br>• Change in TDI focal score and SGRQ total score at Week 52<br>• Time to first moderate or severe COPD exacerbation<br>• Daily rescue medication use over 52 weeks
GLOW5 (Chapman et al. 2014: Study number NCT01613326)	12 weeks<br>N=657	Glycopyrronium 50 µg od<br>Tiotropium 18 µg od (blinded)	• Aged ≥40 years<br>• Smoking history ≥10 pack-years	Primary endpoint:<br>• Trough FEV_1 at Week 12 (non-inferiority of glycopyrronium

			• Moderate-to-severe COPD (post-bronchodilator FEV_1 ≥30% and <80% predicted; post-bronchodilator FEV_1/FVC ratio <0.70)	versus tiotropium)<br>Key secondary:<br>• Trough FEV_1 at Week 12 (superiority of glycopyrronium versus tiotropium if non-inferiority demonstrated)<br>• Spirometric outcomes (FVC, peak FEV_1, FEV_1 $AUC_{0\text{-}4h}$, IC)<br>• Change in TDI focal score and SGRQ total score at Week 12<br>• Time to first moderate or severe COPD exacerbation<br>• COPD symptom score<br>• Daily rescue medication use over 12 weeks
SHINE (Bateman et al. 2013: Study number NCT01202188)	26 weeks<br>N=2144	QVA149 (indacaterol 110 µg/glycopyrronium 50 µg) od<br>Indacaterol 150 µg od<br>Glycopyrronium 50 µg od<br>Placebo<br>Tiotropium 18 µg od (open-label)	• Aged ≥40 years<br>• Smoking history ≥10 pack-years<br>• Moderate-to-severe COPD (post-bronchodilator FEV_1 ≥30% and <80% predicted; post-bronchodilator FEV_1/FVC ratio <0.70)	Primary endpoint:<br>• Trough FEV_1 at Week 26 (superiority of QVA149 versus indacaterol and glycopyrronium)<br>Key secondary:<br>• Change in TDI focal score and SGRQ total score at Week 26 (QVA149 versus placebo)<br>• Daily rescue medication use over 26 weeks

				• Trough FEV_1 at Week 26 (QVA149 versus open-label tiotropium)<br>• Effects of each treatment on dyspnea, health status, symptoms, rescue medication use, safety (including cardiovascular), lung function at different timepoints

GLOW, Glycopyrronium bromide in COPD airways clinical study; od, once daily; FEV_1, forced expiratory volume in one second; FVC, forced vital capacity; SGRQ, St George's Respiratory Questionnaire; TDI, transition dyspnea index
